# Determining Deformation Transition in Polyethylene under Tensile Loading

**DOI:** 10.3390/polym11091415

**Published:** 2019-08-28

**Authors:** Na Tan, P.-Y. Ben Jar

**Affiliations:** Department of Mechanical Engineering, University of Alberta, 10-203 Donadeo Innovation Centre for Engineering, 9211-116 Street NW, Edmonton, AB T6G 1H9, Canada

**Keywords:** multi-relaxation test, deformation transition, polyethylene, density, WAXS

## Abstract

The multi-relaxation (MR) test was developed based on the concept that stress relaxation behavior can be used to reflect the material state of polyethylene (PE) under tension. On the basis of this concept, critical stroke for the onset of plastic deformation in the crystalline phase, named the first critical stroke, was determined using the MR test. Results from wide angle X-ray scattering suggest that phase transformation occurred in the crystalline phase of PE after the specimen was stretched beyond the first critical stroke. In this work, the MR test was applied to six PEs of different mass densities to determine their first critical strokes and the corresponding total and quasi-static (QS) stress values. The results show that the first critical stroke had very similar values among the six PEs. More interestingly, the ratio of the QS stress at the first critical stroke to the yield stress from the standard tensile test showed little dependence on PE density. Therefore, it was possible to use the popular short-term tensile test to characterize the critical QS component of the applied stress to initiate plastic deformation in the crystalline phase, which is expected to play a significant role on the long-term, load-carrying applications of PE.

## 1. Introduction

Polyethylene (PE) is a family of commodity polymers with excellent durability, light weight [1,2,3], and relatively low cost. These advantages have attracted applications in many areas, ranging from plastic pipes for water and gas transportation to containers and film for material packaging. For PE used in load-bearing applications, the main concern is around PE’s time-, temperature-, and strain-rate-dependent mechanical properties [4,5,6]. This issue is further complicated by the semi-crystalline nature of PE’s microstructure, which has crystalline and amorphous phases mingled in a lamellar arrangement. Although both phases are involved from the beginning of the deformation process, their role of involvement varies with the applied stress level [5]. A widely accepted concept is that deformation of PE is initially dominated by the amorphous phase, due to its relatively low resistance to deformation [7]. The crystalline phase is involved at this stage through inter-lamellar shear, inter-lamellar separation, and lamellar stack rotation [7,8,9,10], of which the contribution depends on the loading mode and the stress level. In an engineering stress-stroke curve from tensile loading, PE is known to yield at the peak point, at which the lamellar structure starts to disintegrate. Necking occurs after the yielding, which from the microstructural viewpoint is a process that transforms lamellae into fibril clusters.

Strobl and coworkers characterized the mechanism transition in the deformation process discussed above by using four critical strains, of which the first two occurred before the drastic disintegration of the lamellae [5,11,12,13,14]. The first critical strain is suggested to have a value of around 0.04, at which local yielding starts in the crystalline phase. In other words, the first critical point for deformation transition is for the onset of plastic deformation in the crystalline phase. The second critical point, defined by Strobl’s group as the point with the maximum curvature on the true stress-strain curve, represents the onset of the collective slipping in the crystalline phase, which is dominated in PE by the slip system of the (100) plane in the [001] direction [15,16,17,18,19]. Note that the corresponding point in the standard tensile test, which is usually conducted at a constant crosshead speed, is the peak point on the engineering stress-stroke curve.

Following the work discussed above, a mechanical test named the multi-relaxation (MR) test was developed to detect the transition from the amorphous-phase-dominant deformation to the involvement of the crystalline phase in PE. The MR test contains multiple stress relaxation stages at different strokes. In view that a relatively small stroke is required to reach the critical point for the mechanism transition above and that a given specimen geometry stroke and strain at such a small deformation level should follow a one-to-one relationship, stroke is used here, rather than strain, to quantify the deformation level introduced to the specimen. This paper gives details of analysis used to determine the critical stroke for the mechanism transition. Since transition from amorphous-phase-dominant deformation to that involving plastic deformation in the crystalline phase is the first transition detectable by the MR test, this point is denoted as the first critical point hereafter. The analysis presented here is based on a standard viscoelastic model, in which the applied stress (named total stress) is divided into the time-independent, quasi-static (QS) component, and the time-dependent, viscous component. In addition, this paper compares six PEs of different mass densities for their stress and stroke for the first critical point. Wide-angle X-ray scattering (WAXS) was used to examine the change of the crystalline phase in the six PEs, before and after the stroke for when the first critical point is reached. 

## 2. Multi-Relaxation (MR) Test 

The concept for the MR test is similar to that proposed by Hong et al. [14], which is to use the stress relaxation behavior to characterize the material state of PE during the tensile deformation. The transition of deformation mechanisms is detected through the change of stress relaxation behavior. The main difference between the MR test and that used by Hong et al. is that the former uses a single specimen for all stress relaxation stages, while the latter uses multiple specimens, one for each stress relaxation stage. The latter approach was also used in our previous work on PE pipe specimens [20], from which critical deformation for the mechanism transition was found to be consistent with that using MR test on a single specimen [21]. However, the use of a single specimen has the advantage of avoiding inconsistency in the stress decay during stress relaxation, which can be caused by, for example, dimensional inconsistency among specimens. Note that our preliminary study found that, even through waterjet cutting, specimen width may vary up to 3% along the gauge section, and the width variation profile may not be consistent among specimens from the same batch. Moreover, the MR test takes the advantage of the computer control function that is available in the test machine. Therefore, all stress relaxation stages can be conducted with the minimum interference from the operator.

**Figure 1:**
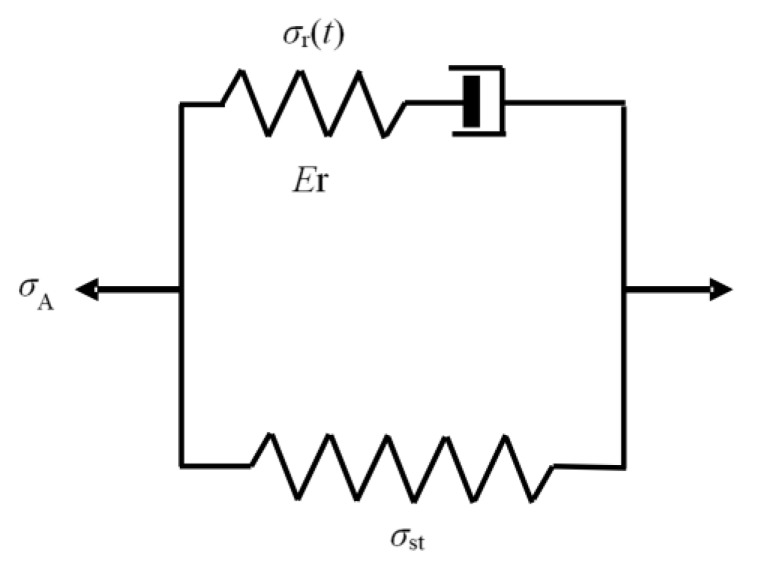
Schematic diagram of the standard, visco-elastic model used in this work.

Analysis of the MR test results is based on a standard, viscoelastic model shown in Figure 1, in which the upper branch represents the time-dependent viscous stress response to deformation and consists of a spring and a damper. The lower branch, on the other hand, represents the QS stress response and contains only a spring. As expressed in Equation (1), the applied stress (*σ*_A_), also referred to as the total stress (*σ*_t_), is the summation of the viscous stress component (*σ*_r_(t)) and the QS stress component (*σ*_st_):

σ_A_ = σ_r_(t) + σ_st_ (1)

According to the model in Figure 1, decay of total stress (∆*σ*_t_) comes from the change of viscous stress (∆*σ*_r_), which is equal to the change of the applied stress during the stress relaxation, as shown in the expression below:

∆*σ*_t_ = ∆*σ*_r_ = *σ*_r_(0) − *σ*_r_(*t*) = *σ*_A_(0) − *σ*_A_(*t*) (2)

where *t* is time measured from the beginning of each stress relaxation stage.

Following the assumption given in [14], the Eyring’s law of viscosity [22,23,24] is adopted to govern the stress response to deformation of the damper:

(3) $$\sigma_{r}/\sigma_{0}=\sinh^{-1}\left({\dot{\varepsilon }}_{D}/{\dot{\varepsilon }}_{0}\right)$$

with

σ_0_ = *KT*/*V* (4)

where *σ*_r_ is the stress applied to the damper, $\dot{\varepsilon }$_0_ the reference strain rate, $\dot{\varepsilon }$_D_ is the strain rate of the damper, *σ*_0_ is the reference stress, *K* is the Boltzmann constant, *T* is the absolute temperature, and *V* is the activation volume. Since two branches of the model in Figure 1 have the same strain and their value should remain constant during the stress relaxation, on the basis of the interaction between the spring and damper in the viscous branch we have

(5) $${\dot{\varepsilon }}_{0}\sinh(\sigma_{r}/\sigma_{0})+{\dot{\sigma }}_{r}/E_{r}=0$$

where *E*_r_ is the modulus for the spring in the viscous branch.

The expression above can also be expressed as

d(*σ*_r_/*σ*_0_)/d*t* = −(*τ*_r_)^−1^ sinh*(σ*_r_/*σ*_0_) (6)

with

(7) $${\left(\tau_{r}\right)}^{-1}={\dot{\varepsilon }}_{0}\;E_{r}/\sigma_{0}$$

where *τ*_r_ is the relaxation time, which, following the work by Hong et al. [14], is given a constant value of 16,000 seconds for all stress relaxation stages. Stress decay (∆*σ*_r_) during the stress relaxation can be derived from Equation (6), as shown in the following expression:

∆*σ*_r_ = *σ*_r_(0) − 2*σ*_0_ tanh^−1^{tanh[*σ*_r_(0)/(2*σ*_0_)] exp(−*t*/*τ*_r_)} (8)

Values for *σ*_r_(0) and *σ*_0_ in Equation (8) were chosen so that the curve generated from the equation matches the stress decay determined from the MR test. Once *σ*_r_(0) and *σ*_0_ values are determined, the *σ*_st_ value can then be calculated from Equation (1). Note that in this work, the *σ*_st_ value is simply an approximation of the real QS stress at a given stroke, since the stress drop did not show any plateau at the end of each stress relaxation stage. Similarly, the corresponding *σ*_r_(0) value is different from that determined from specimens subjected to the single stress relaxation stage, in view that the *σ*_r_(0) value is dependent on the deformation history. Nevertheless, *σ*_r_(0) and *σ*_st_ values determined from these MR tests can be used to indicate the trend of change of these values as functions of stroke. 

**Figure 2:**
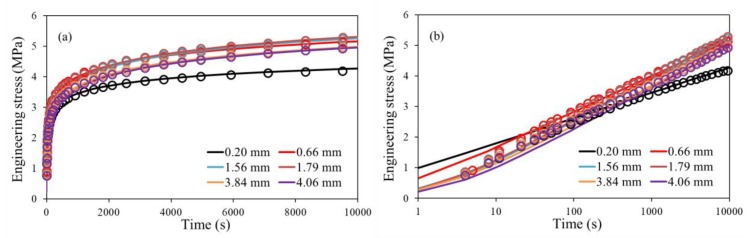
Examples of stress drop obtained from the multi-relaxation (MR) tests (open circles) and generated based on Equation (8) (solid lines) for all stress relaxation stages: (**a**) with time in the linear scale, and (**b**) with time in the logarithmic scale.

Figure 2 presents examples of the best match between the experimental measurements from different stress relaxation stages and the corresponding curves generated from Equation (8). Figure 2a is a plot based on the linear time scale, and Figure 2b on the logarithmic time scale. The legends represent strokes used for the stress relaxation stages. As shown in Figure 2, the curves generated from Equation (8) do not match the experimental measurements for the entire stress relaxation period. Bartczak [25] has suggested to use at least two sets of Eyring’s parameters to fit the experimental measurements for the entire stress relaxation period. In this work, however, only one set of Eyring’s parameters was used for the curve fitting, to match the stress drop mainly in the timeframe above 1000 seconds, as shown in Figure 2b. This is because, firstly, such a curve-fitting process is simple and can be used to determine *σ*_st_ values close to the real QS stresses. Secondly, using one set of Eyring’s parameter has no effects on the relative change of *σ*_0_, which is used to identify the stroke for the first critical point. Through this curve-fitting process, variations of *σ*_0_ and *σ*_st_ are established as functions of stroke. It is worth pointing out that, as shown in Equation (4), variation of *σ*_0_ is independent of the *τ*_r_ value used for the curve fitting. Therefore, the use of *σ*_0_ to determine the stroke for the first critical point can avoid any unwanted influence from the assumption of constant relaxation time on the characterization of stress relaxation behavior at all stages.

## 3. Experimental Details

### 3.1. Materials and Specimen Dimensions

**Figure 3:**
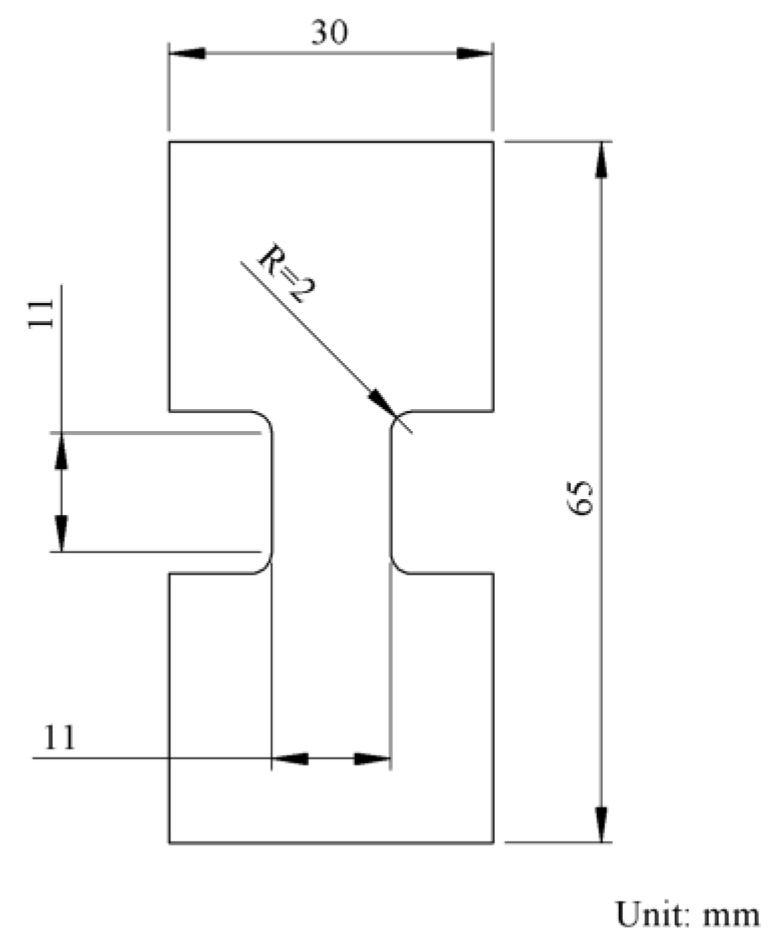
Geometry and dimensions of the modified dog-bone specimens.

Six types of PE were used in the study. As shown in Table 1, these PEs included one linear low-density PE (LLDPE) (#1), and five high-density PEs (HDPE) (#2 to #6), among which density for #2 is close to the lower end of HDPE, #4 close to the upper end, and #3, #5, and #6 in between. Table 1 also provides their yield strength from standard tests, melt index, and co-monomer type. All PEs were in the form of compression-molded plaques of 17.5 × 17.5 cm^2^ in size and 3 mm in nominal thickness, provided by ExxonMobil Chemical. As commercial resins, their molecular weight and molecular weight distribution are not available. Specimens used for the mechanical testing had a modified dog-bone geometry, as depicted in Figure 3, machined from the PE plaques. Choosing such geometry and dimensions in the gauge section was done because of the limited space available in the sample holder of the X-ray diffraction system used for the WAXS experiments. The sufficiently large end tabs were required to maintain the deformation in the gauge section after removing the specimen from the universal test machine. An in-house-designed insert was applied in the gauge section to maintain the deformation during the WAXS experiments, which is described in Section 3.3.

### 3.2. Multi-Relaxation (MR) Test

MR tests were conducted at room temperature using a universal test machine (Qualitest Quasar 100, Lauderdale, FL, USA), with the test program and data acquisition controlled by a personal computer. Crosshead speed for the loading stages was 5 mm/min to introduce a stroke increment of around 0.22 mm, followed by a stress relaxation stage with the stroke fixed. Each stress relaxation stage lasted for 10,800 seconds (3 hours) during which load was recorded as a function of time. Specimens of #4 HDPE fractured at a stroke around 3 mm. All other PEs showed no sign of fracture initiation at strokes above 7 mm, around which the tests were ended. Two specimens were tested for each PE to ensure repeatability of the test results.

### 3.3. Wide-Angle X-ray Scattering (WAXS)

**Figure 4:**
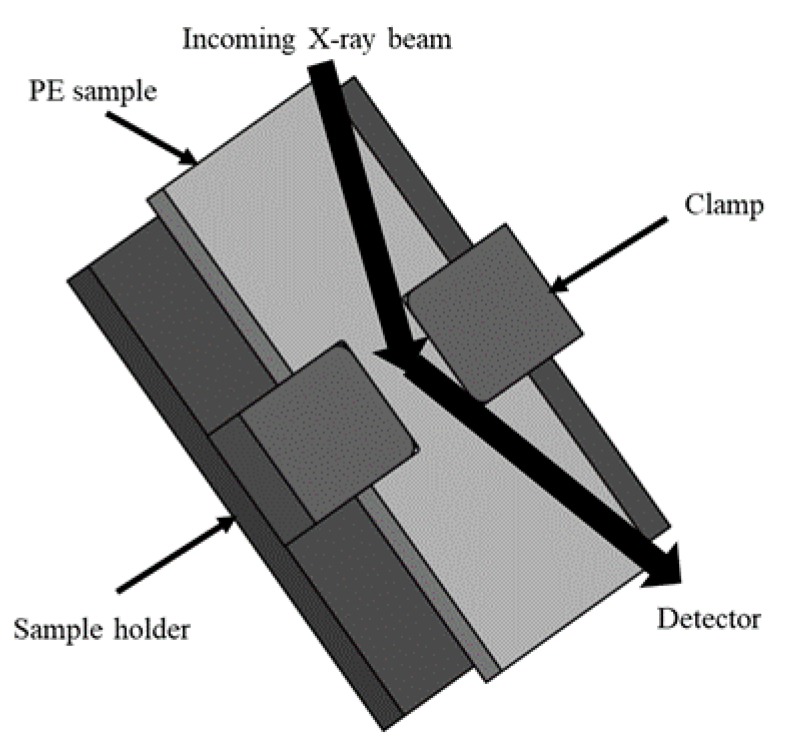
Schematic description of the set-up for the wide-angle X-ray scattering (WAXS) experiment.

One dimensional (1D) WAXS experiments were conducted using a Bruker D8 Discover Diffraction System with Cu-source. Data were collected using a LynxEYE 1-D detector at a scanning speed of 0.8 deg/min, and in the angular range (2θ) from 10 to 64 degrees to cover most of the detectable peaks for PE. Figure 4 gives a schematic diagram of the sample setup for the WAXS experiments.

Each of the specimens for the WAXS experiments were firstly stretched to a pre-determined stroke using the same test set-up as that used for the MR test, except that at the end of the stretch, an in-house-designed and 3D-printed insert was used to maintain the stretch in the gauge section before the specimen was removed from the universal test machine. The assembly of specimen and insert, as shown in Figure 4, was then scanned using an X-ray within 20 minutes after the removal of the specimen from the test machine. Note that as suggested in [26,27], deformation of PE could cause conversion of its lamellar crystals from orthorhombic to monoclinic structures, and the conversion could be reversed if the deformation was removed. Therefore, the insert was used to maintain the deformation level during the X-ray scanning. In this study, the stroke range used in MR tests for the WAXS experiments was to cover the stroke for the first critical point, so that the X-ray spectrum could confirm whether change of the crystalline structure occurred around the first critical point. 

It should be noted that penetration depth of X-ray beam varies with the diffraction angle, especially in the reflection mode shown in Figure 4. The depth variation, in return, affects the collected peak intensity [28]. However, such variation is minimized by comparison of intensity from the diffraction peak at the same angle.

Two specimens from each type of PE were used for the WAXS experiments. Each specimen was used to obtain WAXS spectra at three different strokes, for a total of seven strokes (including the undeformed state). The total time to complete all WAXS spectra for one type of PE was about 9 hours. Therefore, all WAXS tests for each type of PE could be completed in one day. Note that all WAXS spectra presented here already had the machine background removed, which was carried out following the procedure recommended for the machine.

## 4. Results and Discussion

### 4.1. MR Test

**Figure 5:**
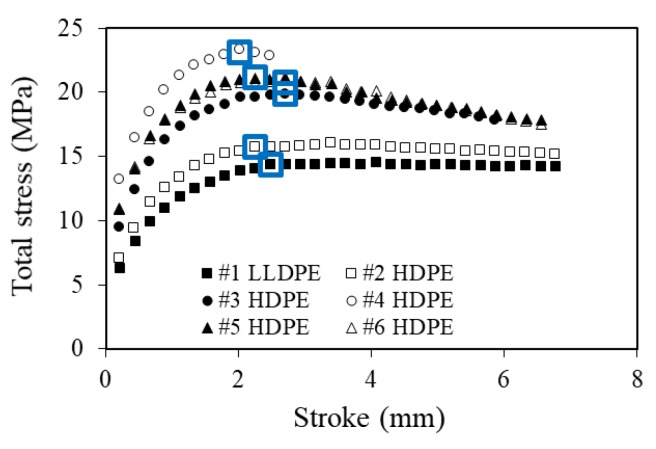
Typical curves from the MR test of total stress versus stroke, with the total stress measured at the beginning of each stress relaxation stage, in which peak points are highlighted using open blue boxes.

Figure 5 presents the typical results from the MR test for the six PEs. Figure 5 summarizes plots of total stress at the beginning of each stress relaxation stage as a function of stroke used for the stress relaxation. The peak point, corresponding to yielding of the specimen, is highlighted in the figure using open blue boxes, all in the range from 2 to 2.7 mm. Some curves, such as that for #2 HDPE, contain a secondary, relatively shallow peak at a stroke above 3 mm. Such a peak is quite common for PEs with side chain branches in the PE molecules. The work of [29,30], based on dynamic mechanical analysis, suggested that the higher the branch density in the PE molecules, the more significant the secondary peak appears. The significance of such a peak is also affected by the cooling process when making plaques from PE pellets.

Note that all PE specimens, except those of #4 HDPE, showed a clear necking behavior at the end of the MR test, at a stroke around 7 mm. As mentioned earlier, the specimens of #4 HDPE had a premature fracture at a stroke around 3 mm, without any indication of neck development.

**Figure 6:**
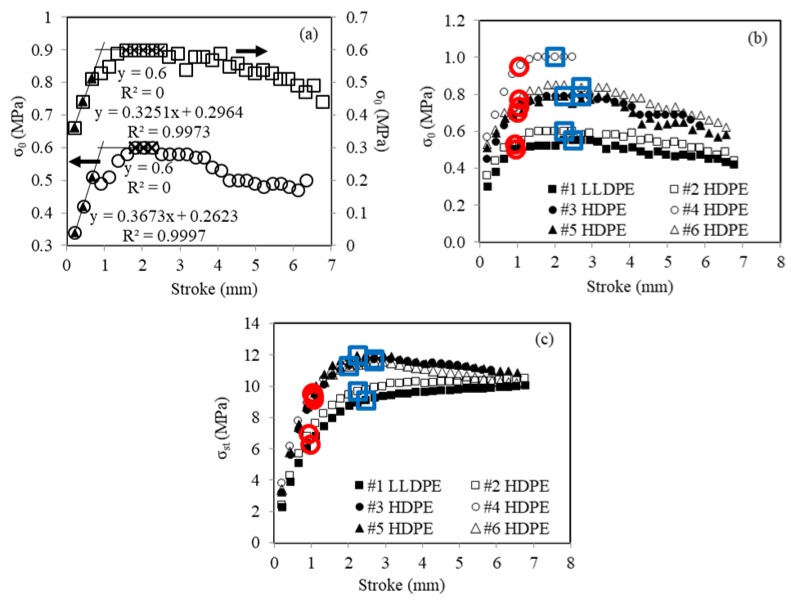
Summary of MR test results: (**a**) sample curves showing the approach used to determine the stroke for the first critical point, (**b**) typical variation of *σ*_0_ with stroke, (**c**) typical variation of *σ*_st_ with stroke (highlighted open red circles and open blue boxes indicate the first critical point and the yield point, respectively).

Data from the stress relaxation stages were analyzed following the curve-fitting process described in Section 2, in which *σ*_r_(0) and *σ*_0_ values were used as fitting parameters to generate a curve based on Equation (8), with *τ*_r_ = 16,000 seconds, to fit the experimentally-measured time function of the stress decay, ∆*σ*_r_. The critical point for the change of trend line in the *σ*_0_-stroke curve was then used to determine the stroke for the first critical point. Figure 6a presents two examples of the *σ*_0_-stroke curves obtained from this study, both from #2 HDPE. The top curve showed a gradual increase of *σ*_0_ with the increase of stroke until a plateau was reached. This was a typical curve for all PEs used in the study. Occasionally, a small drop of *σ*_0_ appeared after its initial linear increase with the increase of stroke, as shown in the bottom curve of Figure 6a. The cause for such a small drop of *σ*_0_ was not clear at this stage, but its appearance did not affect the general trend line of *σ*_0_ versus stroke afterwards. Therefore, stroke for the first critical point was determined based on the intersection of two trend lines, one for the initial linear increase of *σ*_0_ with stroke, and the other for the horizontal line representing the *σ*_0_ value in the plateau region. In this way, possible inconsistency of the critical stroke values due to the presence of a small drop of *σ*_0_ could be avoided. As depicted in Figure 6a, critical strokes from the two curves have very similar values. 

Figure 6b summaries *σ*_0_ stroke plots, one for each PE used in the study. The first critical points are highlighted using open red circles, determined based on the approach depicted in Figure 6a. For comparison, the points corresponding to specimen yielding are also highlighted in Figure 6b using open blue boxes. The data suggest that among the six PEs, strokes for the first critical point have better consistency than those for the yield point. The corresponding *σ*_st_ values at the beginning of each stress relaxation stages are summarized in Figure 6c. Similarly, the first critical points and yield points are highlighted in this figure using open red circles and open blue squares, respectively. It is interesting to point out that, as shown in Figure 6c, peak *σ*_st_ values are only about 50–60% of the peak values of total stress shown in Figure 5, suggesting that even with several stress relaxation stages before reaching the peak point, the applied stress still has a significant viscous stress component. 

### 4.2. WAXS

**Figure 7:**
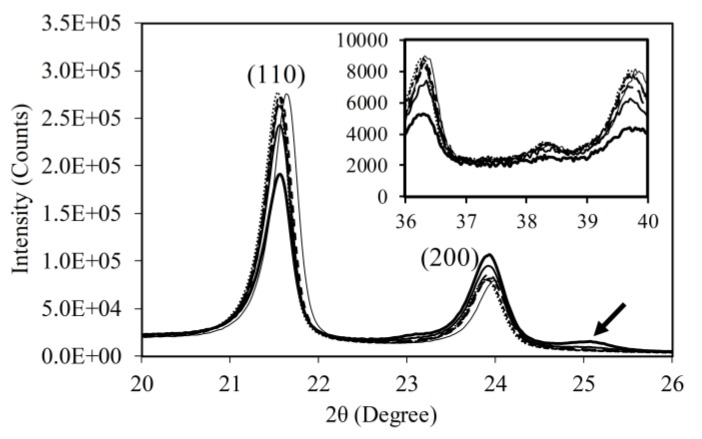
Typical WAXS spectra with different strokes (from #2 HDPE) (peak intensity for (110) decreased with increase of stroke applied to specimens).

Figure 7 presents typical WAXS spectra obtained from PE specimens used in the study. Spectra in the figure are all from #2 HDPE, stretched to different strokes. Two major peaks, labelled (110) and (200), at 2θ of 21.6° and 24.0°, respectively, are from the orthorhombic structure [31,32]. Stroke for each spectrum is not provided in the figure in order to maintain clarity of the curves. The general trend of change among the curves in Figure 7 is that peak intensity for (110) decreased with the increase of stroke, but the opposite trend occurred for (200) (i.e., increase with the increase of stroke). In addition, a minor peak, as indicated by an arrow on the right shoulder of the (200) peak, had peak intensity increase with increase of the stroke above a critical value, which will be discussed later in this section. As suggested in [8,27,33,34,35], growth of this small peak is due to the development of a monoclinic structure that is known to exist in PE under tensile deformation.

The insert in Figure 7 presents a collection of spectra in the 2θ range from 36° to 40°, in which the peak intensity also decreases with the increase of stroke. However, the intensity level for spectra in the insert of Figure 7 was much lower than that for the two major peaks. Furthermore, change of the intensity for peak (200) was less significant than that for peak (110), and work reported in [8,36], for PEs different from those used here, showed little sensitivity of the intensity for peak (200) to the deformation change. Therefore, the following discussion is based on the change of intensity for peak (110) at 2θ of 21.6°.

**Figure 8:**
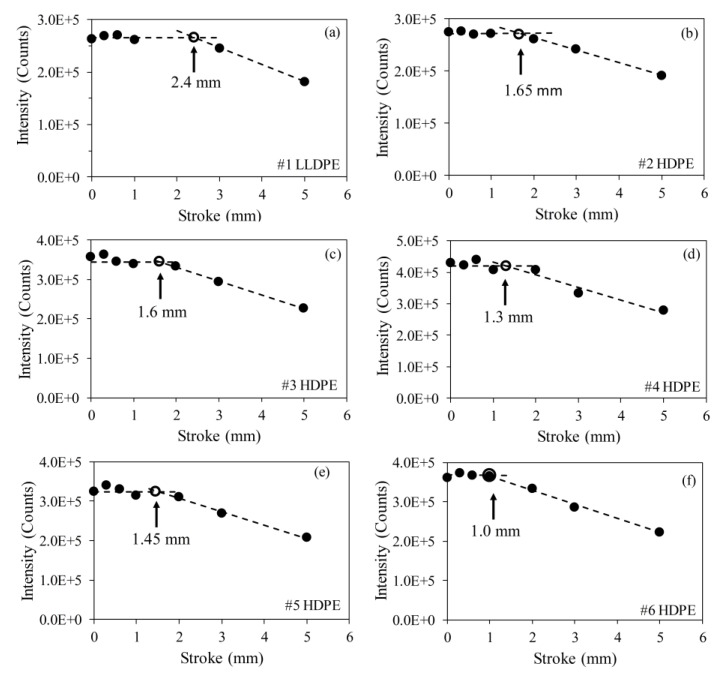
Absolute intensity of peak (110) from the orthorhombic structure of PE, as a function of stroke used to stretch the specimens.

Figure 8 summarizes the intensity variation for peak (110) as a function of stroke used to stretch the specimens. At small strokes, the peak intensity was relatively constant, showing little dependence on the stroke, but when the stroke was sufficiently large, the peak intensity decreased noticeably with the increase of stroke. As a result, Figure 8 indicates that the change of peak intensity went through a transition. Critical stroke for the transition was determined based on the assumption that the peak intensity remained constant below the critical stroke, but above it, decreased linearly with the increase of stroke. The critical stroke for each PE is presented in Figure 8 using an open circle, with the corresponding stroke value given under an arrow that points to the open circle. Figure 8 suggests that the critical stroke for the onset of degradation in the orthorhombic crystalline structure can be detected using change in intensity of peak (110), and that the extent of degradation increased with the increase of stroke. However, Figure 8 suggests significant variation of the critical stroke values for the six PEs, which is different from that shown in Figure 6 (represented by open red circles).

**Figure 9:**
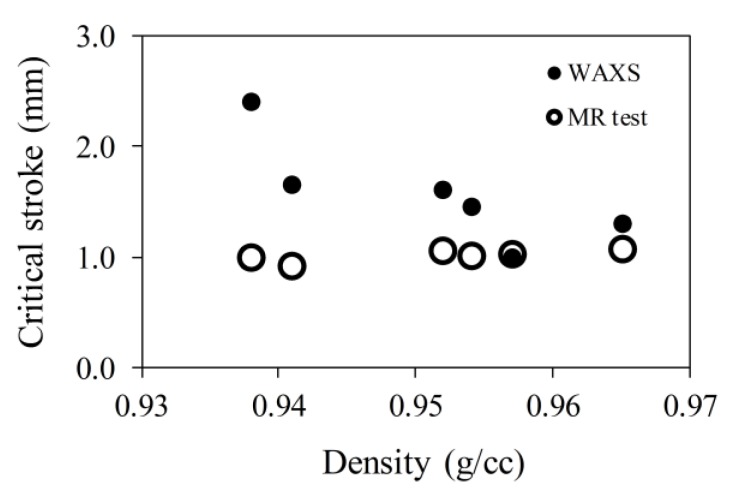
Comparison of critical strokes in Figure 8 and those for the first critical point from the MR test.

To resolve the issue of different critical stroke values given in Figure 6; Figure 8, critical strokes determined from the WAXS spectra and those from the MR tests are summarized in Figure 9, plotted as a function of PE density. Note that PE density is used here as a material parameter for convenience. Other parameters, such as degree of crystallinity from WAXS spectra [7,37], could also be used to represent material characteristics. However, our analysis has shown that values for the degree of crystallinity were linearly proportional to the values for PE density. Therefore, trend line of the dependence of test results on PE density should be the same as that on degree of crystallinity. In the following discussion, the former parameter was selected as the material parameter.

Figure 9 suggests that the critical strokes from the WAXS experiment were either larger than or equal to (for #6 HDPE, with PE density of 0.957 g/cc) the stroke for the first critical point from the MR test. Note that stroke used in Figure 8 represents the dimension of the insert used to maintain the specimen deformation during the WAXS experiments. However, the maximum stroke required to place the insert in the specimen was always larger than the insert dimension, and the amount of extra stroke required varied among the PE specimens. Therefore, critical stroke values determined from Figure 9 may have a bigger uncertainty than that determined from the MR test for which a single specimen was used, with stroke resolution of the test machine being better than 0.01 mm. Since Figure 9 suggests that none of the critical stroke values from the WAXS spectrum were smaller than that determined from the MR test, it is believed that critical stroke values determined from the MR test represented the lower bound of the possible critical stroke values determined from the WAXS spectrum, and that the critical stroke values from the MR test were more reliable than those from the WAXS spectrum for quantifying the deformation level required to start plastic deformation in the crystalline phase of PE. Nevertheless, the WAXS experiments provide plausible evidence to support the fact that the stroke for the first critical point, determined from the MR test, represented the onset of plastic deformation in the crystalline phase of PE.

### 4.3. Discussion

Using the standard visco-elastic model shown in Figure 1, total stress, *σ*_t_, applied to PE in the MR test can be divided into the time-independent component, *σ*_st_, and the time-dependent component, *σ*_r_(t). On the basis of the change of the trend line for *σ*_0_, critical strokes for the local and global plastic deformation of the crystalline phase, i.e., the first critical point and the yield point, respectively, were identified, and their corresponding *σ*_st_ values were determined.

**Figure 10:**
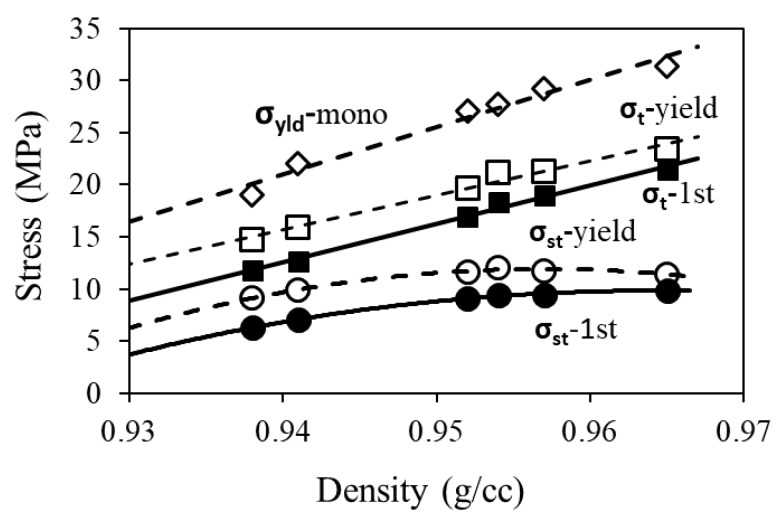
Summary of *σ*_st_ and *σ*_t_ values at the first critical point and yield point from the MR tests (*σ*_st_-1st, *σ*_st_-yield, *σ*_t_-1st, and *σ*_t_-yield), and yield strength listed in Table 1 (*σ*_yld_-mono), plotted as functions of PE density.

Figure 10 depicts plots of *σ*_t_ (squares) and *σ*_st_ (circles) at the first critical point (solid symbols) and yield point (open symbols) determined from the MR tests, and yield strength values listed in Table 1 (*σ*_yld_-mono, open diamonds), which were determined using a standard test. The figure shows that all stress values that include viscous and quasi-static components, i.e., *σ*_yld_-mono, *σ*_t_-yield, and *σ*_t_-1st, show a relatively linear relationship with PE density. However, the corresponding *σ*_st_ values determined from the MR test, i.e., *σ*_st_-yield and *σ*_st_-1st, show a nonlinear relationship with PE density. 

As shown in Figure 10, the linear relationship between *σ*_t_ at the yield point and PE density was similar to that for *σ*_yld_-mono, which is consistent with the common belief that yield strength for PE follows a close to linear relationship with PE density [38]. Such a linear relationship also existed for *σ*_t_ at the first critical point, as shown by the curve of *σ*_t_-1st in Figure 10. However, the figure also suggests that the relationship between *σ*_st_ and PE density was non-linear, at both the first critical point and the yield point. This could be caused by the assumption of the constant *τ*_r_ value of 16,000 sec in the Eyring’s model for the entire deformation process, as the *σ*_st_ value required for Equation (8) to fit the experimental curve is known to vary with the *τ*_r_ value. Therefore, further investigation to allow *τ*_r_ to vary with stroke is needed to verify whether the relationship between *σ*_st_ and PE density is indeed non-linear, as suggested by Figure 10. Nevertheless, using constant *τ*_r_ was sufficient for our purpose at this stage. Our on-going study is to find a way to make *τ*_r_ as a free parameter in the curve fitting process.

**Figure 11:**
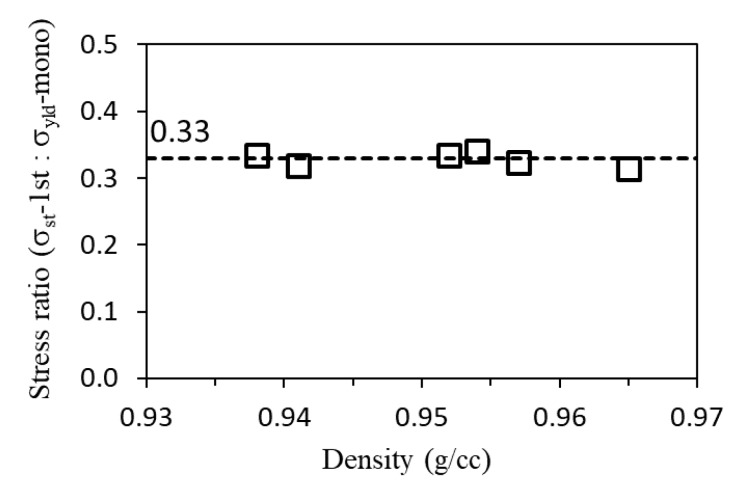
The ratio of *σ*_st_ at the first critical point from the MR test (*σ*_st_-1st) to yield stress from the standard tensile test (*σ*_yld_-mono) listed in Table 1, plotted as a function of PE density.

In addition to the nonlinear relationship between *σ*_st_ and PE density, this study also discovered a possible linear relationship between stress ratio and PE density. Figure 11 presents the stress ratio of *σ*_st_ at the first critical point (*σ*_st_-1st) to the yield strength listed in Table 1 (*σ*_yld_-mono), plotted as a function of PE density. The figure suggests that this stress ratio was nearly constant among six PEs used in the study, showing little dependence on the PE density. This phenomenon sheds a light on the possibility of using a short-term test to determine the time-independent stress component, *σ*_st_, which at this stage cannot be correctly measured using any standard test. 

In view that *σ*_st_-1st represents the critical QS stress to initiate local plastic deformation in the crystalline phase of PE, its value could play a significant role on the long-term performance of PE, especially for load-carrying applications. The possibility of using a short-term test to determine the *σ*_st_-1st value, as indicated in Figure 11, will greatly benefit the industry for a quick evaluation of the long-term performance of PE. Developing a study to address the issues discussed above is important to confirm such a possibility, and is planned for once this manuscript is prepared.

## 5. Conclusions

A new multi-relaxation (MR) test method was developed and applied to six PEs of different densities to determine their critical points for the transition from the amorphous-phase-dominant deformation to the involvement of the crystalline phase. The critical stroke level for the onset of local plastic deformation in the crystalline phase, named the first critical point, was identified for the six PEs. These critical stroke values were found to be nearly constant and independent of the PE density. All of these phenomena were consistent with those reported in the past, using similar analyses but based on test results from multiple specimens [14,20]. WAXS experiments were conducted to examine the change of the crystal structure at the first critical point. The WAXS spectra confirmed that after reaching the stroke for the first critical point, intensity for the (110) peak showed a significant decrease with the increase of stroke.

Through the study on six PEs, it was discovered that the *σ*_st_ component for the first critical point did not show a linear relationship with PE density, in contrast to the yield stress measured from the standard test.

This study also discovered that the stress ratio of *σ*_st_-1st to *σ*_yld_-mono from the standard test showed a nearly constant value, independent of PE density. Since the long-term, load-carrying performance of PE should be based on its *σ*_st_ value, the nearly constant ratio of *σ*_st_-1st to *σ*_yld_-mono indicated the possibility of using short-term tests to evaluate PE’s long-term performance, however, further investigation is needed.

Overall, the study provides some insights on the time-independent mechanical properties of PE and the associated mechanisms for deformation. We intend to apply the information to the evaluation of PE, in order to explore the practical benefits of the MR test on characterization of PE’s long-term, load-carrying performance.

## Figures and Tables

**Table 1:** Material characteristics of polyethylenes (PEs) used in the study. LLDPE = linear low-density PE, HDPE = high-density PE.

Material	Density (g/cc)	Yield Strength (MPa)	Melt Index (g/10 min) at 190 °C/2.16 Kg	Molecular Weight Distribution	Co-Monomer
#1 LLDPE	0.938	19.0	3.3	Unimodal	Hexene
#2 HDPE	0.941	22.0	2.0	Unimodal	Hexene
#3 HDPE	0.952	27.1	6.7	Unimodal	Hexene
#4 HDPE	0.965	31.4	8.2	Unimodal	-
#5 HDPE	0.954	27.7	0.3	Unimodal	Butene
#6 HDPE	0.957	29.2	0.46	Bimodal	Hexene
